# In Situ Biofilm Monitoring Using a Heat Transfer Sensor: The Impact of Flow Velocity in a Pipe and Planar System

**DOI:** 10.3390/bios15020093

**Published:** 2025-02-06

**Authors:** Andreas Netsch, Shaswata Sen, Harald Horn, Michael Wagner

**Affiliations:** 1 DVGW Research Center at the Engler-Bunte-Institut, Engler-Bunte-Ring 9a, 76131 Karlsruhe, Germany; harald.horn@kit.edu; 2 Engler-Bunte-Institut, Water Chemistry and Water Technology, Karlsruhe Institute of Technology (KIT), Engler-Bunte-Ring 9a, 76131 Karlsruhe, Germanymichael.wagner@kit.edu (M.W.); 3 Institute for Biological Interfaces 1 (IBG-1), Institute for Biological Interfaces, Karlsruhe Institute of Technology (KIT), Hermann-von-Helmholtz-Platz 1, 76344 Eggenstein-Leopoldshafen, Germany

**Keywords:** heat transfer, biofilm sensor, hydrodynamics, bioelectrochemical system, mathematical model

## Abstract

Industrially applied bioelectrochemical systems require long-term stable operation, and hence the control of biofilm accumulation on the electrodes. An optimized application of biofilm control mechanisms presupposes on-line, in-situ monitoring of the accumulated biofilm. Heat transfer sensors have successfully been integrated into industrial systems for on-line, non-invasive monitoring of biofilms. In this study, a mathematical model for the description of the sensitivity of a heat transfer biofilm sensor was developed, incorporating the hydrodynamic conditions of the fluid and the geometrical properties of the substratum. This model was experimentally validated at different flow velocities by integrating biofilm sensors into cylindrical pipes and planar mesofluidic flow cells with a carbonaceous substratum. Dimensionless sensor readings were correlated with the mean biovolume measured gravimetrically, and optical coherence tomography was used to determine the sensors’ sensitivity. The biofilm sensors applied in the planar flow cells revealed an increase in sensitivity by a factor of 6 compared to standard stainless steel pipes, as well as improved sensitivity at higher flow velocities.

## 1. Introduction

The field of applications for bioelectrochemical systems (BESs) is wide, utilizing various substrates/waste streams to produce value-added products, including energy-efficient waste water treatment [1] with the use of microbial fuel cells (MFCs), or the production of hydrogen in microbial electrolysis cells (MECs) [2]. Common denominator among BESs are electroactive biofilms growing on the anode coupling the metabolic conversion of organic substrates by anodic bacteria with extracellular electron transfer (EET) to a solid electrode. The morphology of the electroactive biofilm on the anode is of central importance to the efficiency and functionality of BESs [3,4]. The goal of upscaling BESs faces a number of challenges in the transition from laboratory-sized reactors to pilot- or industrial-scale systems [5,6], among which is efficient mass transfer between the bulk phase and the electroactive biofilm at the electrode–biofilm interface. In detail, in an MFC, organic carbon diffuses towards the anode, where it is oxidized by electroactive bacteria to CO_2_ and H^+^. The products need to diffuse out of the biofilm into the bulk phase. Otherwise, subsequent substrate gradients [7,8,9] or acidic pH environments [8,10] in the biofilm can limit or inhibit the electroactive bacteria in their current production. An additional bottleneck of transport phenomena, which also hampers the anodic efficiency of BESs, is the reported limited distance of electron transfer from bacteria to the electrode by cytochromes, electron shuttles/mediators, or conductive pili [11,12,13]. The combination of limitations deriving from extracellular electron transfer distance and diffusion resistance, along with the need for a high quantity of electroactive bacteria, suggests an optimal biofilm thickness range for electroactive biofilms. Recently reported optimal biofilm thicknesses for electroactive model organisms range from a few µm for *Shewanella* sp. [14], to approx. 10 µm for *Desulfuromonas acetexigens* [15] and up to 20–100 µm for *Geobacter sulfurreducens* [3,16,17].

With the aim of long-term, stable bioelectrochemical processes in reactor systems utilizing waste streams, the control of biofilm thickness within an optimum range would support more efficient applications [18]. Therefore, continuous monitoring of the state of the electroactive biofilm by means of on-line sensors on the anodes is desirable for the purpose of an industrially usable process control. Industrially applicable biofilm sensors require an on-line, in situ and non-invasive characterization of the biofilm to provide relevant information regarding the biofilm’s morphology [19]. While most of the reported methods of biofilm monitoring have been investigated in lab-scale applications, a few industrially applicable sensors, including electrochemical sensors (e.g., ALVIM [20], BioGeorge [21], BIOX [22]), mechanical sensors (Solenis OnGuard [23]), optical sensors (OPTIQUAD [24]) and thermal sensors (DEPOSENS^®^ [25,26]), are known. An extensive discussion of the advantages and limitations of the respective biofilm sensors or sensing methods can be found in the reviews by Janknecht and Melo [27], Nivens et al. [28] or Pereira and Melo [19].

Thermal biofilm sensors, which utilize the additional thermal resistance of deposits (such as biofouling or scaling) that increases linearly with accumulation in the thickness of the deposits [27], can be applied without intruding into the flow channel, and thereby are electrically isolated from the electrode [25]. Limiting factors for thermal biofilm sensors have been reported to be a relatively low sensitivity, as well as a lack of differentiation of the chemical nature of the deposit [19,27]. Recently, Netsch et al. [25] demonstrated the applicability of heat transfer biofilm sensors to carbonaceous material, in this case, a compound material of graphite–polypropylene (C-PP). Electrodes of BESs are commonly made from carbonaceous materials, due to their good electrical conductivity, low costs and biocompatibility [2,29]. However, the increased thermal conductivity of the substratum material in reference to the commercially available application, which was stainless steel (SST), resulted in a loss of sensitivity of 80% compared to SST, thereby diminishing the precision of the substratum material’s applicability to BESs, as the optimal thickness is in the range of 50–100 µm. Boukazia et al. [30] showed an improvement in the metrological performance of a heat transfer sensor with steady thermal excitation, using a PVC scotch as a model deposit, when applied to a planar surface in comparison to a curved sensor surface intruding into the medium.

This study investigates the sensitivity of the DEPOSENS^®^ heat transfer biofilm sensor, targeting an application in BES reactors. To achieve this, the sensor was applied to mesofluidic flow cells with a planar C-PP substratum and a curved substratum in SST pipes (standard configuration), in which waste water biofilms were cultivated at different flow velocities, as heat transfer is highly dependent on hydrodynamic conditions. The dimensionless output of the sensor (arbitrary units, a.u.) was correlated with the biofilm thickness through detailed analysis of biofilm parameters by means of optical coherence tomography (OCT), for the determination of the sensor sensitivity. This approach validates the sensor’s capability to continuously monitor the biofilm thickness, and identifies the impact of substratum geometry and hydrodynamic conditions on the sensor’s sensitivity.

## 2. Materials and Methods

### 2.1. Integration of Biofilm Sensors in Mesofluidic Flow Cells

Within this study, DEPOSENS^®^ biofilm sensors, manufactured by Lagotec GmbH (Magdeburg, Germany), were investigated, which have been applied commercially to stainless steel pipes for the monitoring of deposits in cooling systems, in the paper industry (www.lagotec.de accessed: 1 July 2024) or for membrane fouling surveillance [26]. Netsch et al. [25] have recently demonstrated the application of these sensors to other materials, in particular a carbon–polypropylene (C-PP) compound material, despite its increased thermal conductivity (*λ_C-PP_* = 21 W/(m × K)) compared to that of stainless steel (*λ_SST_* = 13.3 W/(m × K)). Briefly, the working principal of the sensor is based on a reduction in the heat transfer due to accumulating deposits with a lower thermal conductivity than the substratum, e.g., biofilms (*λ_Biofilm_* = 0.6 W/(m × K)) [31]. A constant temperature difference (Δ*T* = 10 K) is set between the heater on the sensor and the medium temperature. The sensor signal results from the heat flux $\dot{Q}$, in reference to the heat flux at a zeroed value (clean state of the substratum), at a constant flow velocity. The measurement interval was 5 min. For a more detailed description of the sensor, the authors refer to Netsch et al. [25]. The biofilm sensors were applied to two pipes with an identical inner diameter (*d_i_* = 25.4 mm), made of SST (*l_SST_* = 250 mm) and C-PP (*l_C-PP_* = 300 mm), respectively.

**Figure 1:**
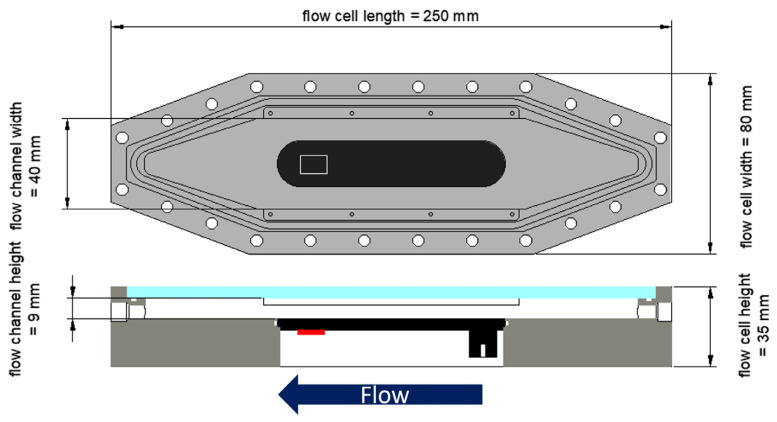
The top view and cross-section of the mesofluidic flow cell. The white rectangle marks the OCT imaging area (4 × 6 mm^2^) at the point of measurement of the biofilm sensor. In the cross-section, the biofilm sensor (red) is glued to the C-PP substratum (black), without contact with the bulk medium. The flow channel of the flow cell has a cross-section of 40 × 9 mm^2^ and is closed by an optical window (light blue). The direction of flow is from right to left.

Additionally, a mesofluidic flow cell (see Figure 1), developed by Hackbarth et al. [32] (flow channel: W × H = 40 × 9 mm^2^), made of polyoxymethylene (POM) was modified by integrating a C-PP substratum (L × W × H = 101 × 20 × 4 mm^3^). In all cases, the same biofilm sensor was installed on the outside of the SST pipes, the C-PP pipes and the bottom of the C-PP substratum in the flow cell, respectively, and enclosed by polyurethane (PUR) thermal insulation.

### 2.2. Experimental Setup

**Figure 2:**
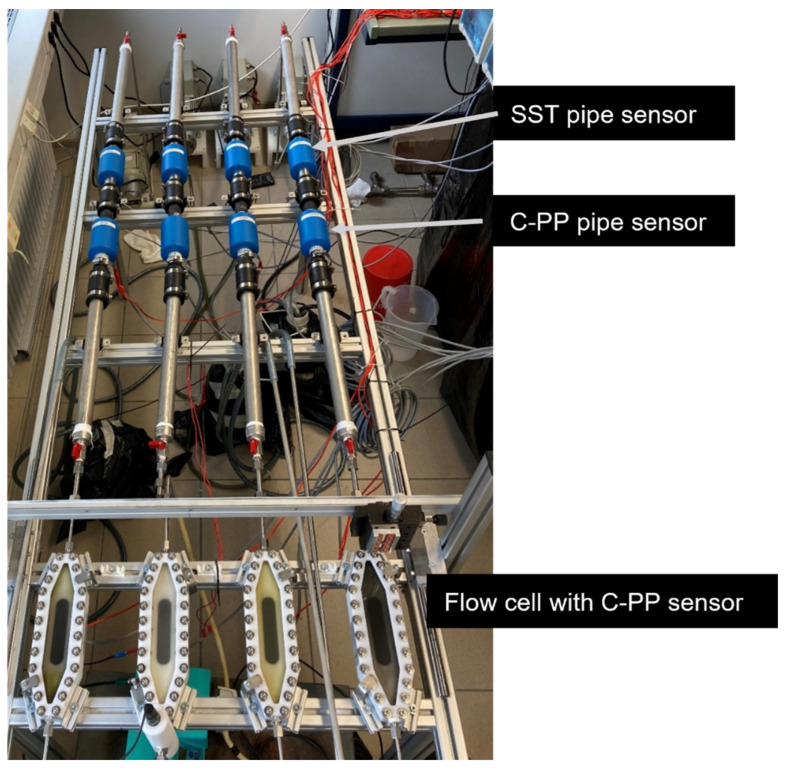
A photograph of the experimental setup, showing the four replicates operated in parallel. Each replica was equipped with one SST pipe, one C-PP pipe and a mesofluidic flow cell with a planar C-PP substratum. Biofilm sensors were integrated into each of the three systems (compare Figure 1). The direction of flow was from top to bottom.

The biofilm cultivation experiments were performed in a recirculatory system. Four parallel lines, each with an SST pipe biofilm sensor, a C-PP pipe biofilm sensor and a mesofluidic flow cell with an integrated biofilm sensor, were installed in series on a mounting platform with a movable holder for the OCT probe (see Figure 2). Each line was provided with a magnetic gear pump (Niemzik PAT, Haan, Germany) to recirculate the cultivation medium through the system from a 5 L reservoir. Before each experiment, all parts of the system were thoroughly cleaned manually to remove any residual deposits. This was confirmed by means of OCT in the flow cells. Due to the cultivation of a waste water biofilm, it was decided not to perform pre-experimental sterilization or operation under sterile conditions. Within this study, four different flow rates were investigated. Table 1 gives an overview of the performed experiments with their respective parameters and number of replicates.

The parallel lines of the system were inoculated with 5 L of the supernatant of activated sludge from a local waste water treatment plant. During the first 24 h of inoculation, the flow velocity was reduced to 50% of the experimental flow velocity. Subsequently, an artificial cultivation medium based on unsterile tap water, with a molar C:N:P ratio of 100:10:1, was added to the system to provide optimal conditions for anaerobic cultivation. Sodium acetate (c = 238.46 mg/L) was provided as a carbon source (chemical oxygen demand (COD) = 200 mg/L), and ammonium chloride (c(NH_4_Cl) = 31.1 mg/L) as a nitrogen source. A phosphate buffer was used to adjust the pH to medium, at 7.2. The cultivation medium for each line was sampled individually on a regular basis, and COD, NH_4_^+^-N, and PO_4_^3−^-P were measured by a Hach vial test. To avoid substrate limitations, the medium was replenished if its substrate contents decreased below a threshold of COD <20 mg/L or NH_4_^+^-N < 1 mg/L.

### 2.3. Biofilm Quantification and Structural Parameters

Biofilm accumulation in the mesofluidic flow cells was monitored daily, by means of optical coherence tomography (OCT), for in situ and non-invasive analysis of the biofilm’s morphological parameters on the C-PP substratum. A GANYMEDE spectral domain OCT system (GAN610, Thorlabs GmbH, Dachau, Germany) with a LSM04 objective lens (Thorlabs GmbH, Dachau, Germany) was used. Three-dimensional OCT datasets (C-scans) were acquired at the position of the biofilm sensor (see Figure 1). The imaging volume was set to L × W × H = 4 × 6 × 2.14 mm^3^, with pixel resolutions of 8 µm/px laterally (xy-plane) and 2.06 µm/px axially. Biofilm parameters to describe the morphology were calculated using macros made in-house, operated by ImageJ (version 1.54). Six parameters were determined for the characterization of the biofilm structure, according to the works of Wagner and Horn [33] and Murga et al. [34].

The mean biovolume ${\bar{BV}}_{OCT}$ is defined as the biofilm volume $V_{F}$ (sum of volume of all biofilm voxels) per imaging area $A_{OCT}$.

(1) $${\bar{BV}}_{OCT}=\frac{V_{F}}{A_{OCT}}\;\;({\mu m}^{3}/{\mu m}^{2})$$

The mean biofilm thickness ${\bar{L}}_{F,OCT}$ gives the height of the bulk–biofilm interface to the substratum, thereby taking the cavities and spatial distribution of the biofilm into account. $L_{F,i}$ is the locally measured biofilm thickness, with *N* being the number of pixels.

(2) $${\bar{L}}_{F,OCT}=\frac{1}{N}\sum_{i=1}^{N}L_{F,i}\;\;(\mu m)$$

The substratum coverage (*SC*) describes the fraction of the imaging area $A_{OCT}$ of the substratum that is covered with biofilm.

(3) $$SC=\frac{A_{OCT}-A_{uncovered}}{A_{OCT}}\;\;(\%)$$

The intrinsic porosity Φ measures the volume fraction of voids (*voxel (0)*) beneath the bulk–biofilm interface ${\bar{L}}_{F,OCT}$.

(4) $$\Phi =\frac{\sum voxel\;(0)}{{\bar{L}}_{F,OCT}\times A_{OCT}}\;\;(\%)$$

Both the roughness (*R_a_*) and roughness coefficient (R_a_^*^) describe the surface of the biofilm at the bulk–biofilm interface. The roughness coefficient (R_a_^*^) normalizes the roughness (*R_a_*) to the mean biofilm thickness ${\bar{L}}_{F}$, and allows for a comparison of biofilms with different thicknesses.

(5) $$R_{a}=\frac{1}{N}\sum_{i=1}^{N}\left|L_{F,i}-{\bar{L}}_{F}\right|\;\;(\mu m)$$

(6) $$R_{a}^{*}=\frac{1}{N}\sum_{i=1}^{N}\frac{\left|L_{F,i}-{\bar{L}}_{F}\right|\;}{{\bar{L}}_{F}}\;\;(-)$$

At the end of the experiment, the biofilm in the pipe sensors (C-PP and SST) was measured gravimetrically. Pipe sensors were placed in a vertical position and water was released. Unbound water was drained for 10 min, before the biofilm was scraped off and weighed (KB 2400-2N, Kern & Sohn GmbH, Balingen, Germany). The mean biovolume distributed over the entire area of the pipe sensor (*A_pipe,SST_* = 126 cm^2^, *A_pipe,C-PP_* = 152 cm^2^) was determined according to Equation (7), with *m_biofilm_* describing the mass of the wet biofilm, and $\rho_{biofilm}$ describing the density of the biofilm.

(7) $${\bar{BV}}_{grav}=\frac{m_{biofilm}}{\rho_{biofilm}\times A_{Pipe}}\;\;({\mu m}^{3}/{\mu m}^{2})$$

The gravimetrically determined biovolume can be compared to the biovolume obtained by means of OCT imaging in the mesofluidic flow cells [33].

### 2.4. Determination of Sensor Sensitivity and Statistical Analysis

The dimensionless sensor signal *D*, in arbitrary units (a.u.), requires transfer into a structural biofilm parameter (e.g., mean biovolume $\bar{BV}$) for its implementation as a biofilm monitoring device. The sensor signal was correlated with the mean biovolume acquired from OCT images to develop a linear calibration curve for the sensor in the flow cells at different velocities. The slopes of the calibration curves were inverted to describe the experimentally determined sensitivity of the sensor (compare Equation (8)). A sensor sensitivity with a small value is the most desirable, as this will allow for a more precise distinguishment between biofilms of different thicknesses.

(8) $$sensor\;sensitivity=\frac{1}{slope}=\;\frac{∆\bar{BV}}{∆D}\;({\mu m}^{3}/({\mu m}^{2}\times a.u.))$$

To determine if triplicate experiments suffice for a precise calibration curve, the experiments 2 a–d (see Table 1) were performed as multiple replicates of one flow velocity (u_exp_ = 12 cm/s), and statistically evaluated. A total of 14 individual flow cell runs were investigated. For a comparable evaluation of the resulting calibration curves, the 14 datasets from the replica experiments were randomly recombined as sets of three. This resulted in a total of 364 combination possibilities, for all of which linear regression curves were calculated to identify the respective sensor sensitivity for each flow cell combination. The resulting sensor sensitivities were tested for outliers using Grubb’s Test (*p* < 0.05), to determine the reproducibility of the sensor measurement. This allowed for a comparison with the experiments (1, 3 and 4), with three replicas each.

## 3. Results and Discussion

**Figure 3:**
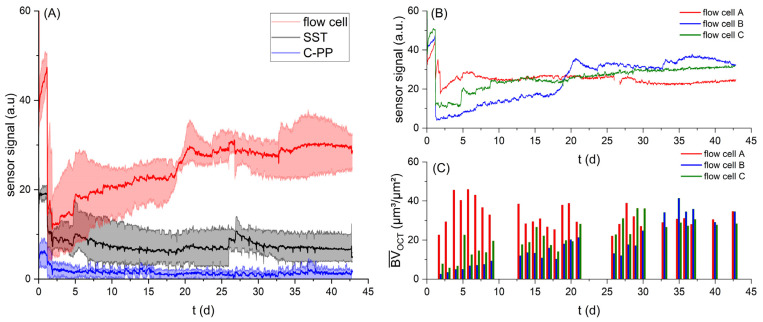
(**A**) The development of the biofilm sensor signal over the cultivation period (t = 45 d) for the sensors integrated in the flow cells (red), SST pipes (black) and C-PP pipes (blue), at a volumetric flow rate of 2.6 L/min (Exp. 2). The mean values are displayed as the thick curve between the minimum and maximum values for the triplicates, respectively. (**B**) The development of the sensor signal and (**C**) mean biovolume (bottom) individually for the three replicate flow cells (**A**–**C**) in Exp. 2.

The integration of the biofilm sensor into the mesofluidic flow cells allowed for continuous non-destructive monitoring of the biofilm accumulation ${\bar{BV}}_{OCT}$ on the substratum directly above the sensor. This allowed for a more detailed observation of the impact of accumulating biofilm on the sensor signal. On the contrary, the biofilm sensors incorporated into the SST and C-PP pipes only allowed for destructive gravimetrical determination of the biovolume ${\bar{BV}}_{grav}$ at the end of the experiment. For comparability with previous investigations presented by Netsch et al. [25], in experiment 2 (compare Table 1), a linear flow velocity in the flow cell of u_exp_ = 12 cm/s (volumetric flow rate of 2.6 L/min) was investigated. Figure 3A shows the development of the sensor signal of the sensors integrated into the flow cells on the C-PP substratum, as well as integrated into the C-PP pipes and SST pipes.

A reduced flow velocity (u_inoc_ = 0.5 u_exp_) was applied during the inoculation to support bacterial attachment in the early stages of the experiment, resulting in an initially increased sensor signal compared to the reference value set for the clean sensors at u_exp_. On Day 2, the sensor signals dropped from increased values during inoculation, with a reduced flow rate for all of the different sensor applications. The sensor signals at Day 2 of the cultivation displayed values of 5–18 a.u. for the flow cell sensor, 6–10 a.u. for the SST sensor and 0–4 a.u. for the C-PP sensor. Large differences in signal development in the flow cells could be seen due to variation in the initial biofilm accumulation at the imaging area in the flow cell, as flow cell A (red) showed the highest biovolume accumulation at Day 2 (23 µm^3^/µm^2^), followed by flow cell C (green) with 8 µm^3^/µm^2^ and flow cell B (blue) with 3 µm^3^/µm^2^. 

Over the course of the cultivation period (t = 45 d), the sensor signal of the flow cell sensors increased to a maximal signal of 27–35 a.u., in contrast to the sensor signals of the sensors integrated into the SST and C-PP pipes, where the sensor signals displayed no markable change from the initial value after the adjustment of the flow rate.

The increase in the sensor signal of the flow cell sensors can be aligned with the increase in detected biovolume in the flow cells (Figure 3B,C). With increasing biofilm growth, the deposits on the substratum above the sensor in the flow cells led to an increase in thermal resistance, measured by the biofilm sensor and converted to a sensor signal in arbitrary units (a.u.). At the end of the cultivation period, OCT C-scans showed a mean biovolume of 35 µm^3^/µm^2^ in flow cells A and B, while in flow cell C, a mean biovolume of 28 µm^3^/µm^2^ was measured. In contrast, the gravimetrical determination of the biofilm accumulated in the SST and C-PP pipes showed a mean biovolume of 118–173 µm^3^/µm^2^ and 261–379 µm^3^/µm^2^, respectively. The larger sensor signal measured by the sensors integrated into the flow cells at smaller accumulated biovolumes indicated an improved performance of the biofilm sensors applied on a planar substratum, compared to on the curved substrata of the SST and C-PP pipes.

### 3.1. Correlation of Sensor Signal with Accumulated Biovolume

**Figure 4:**
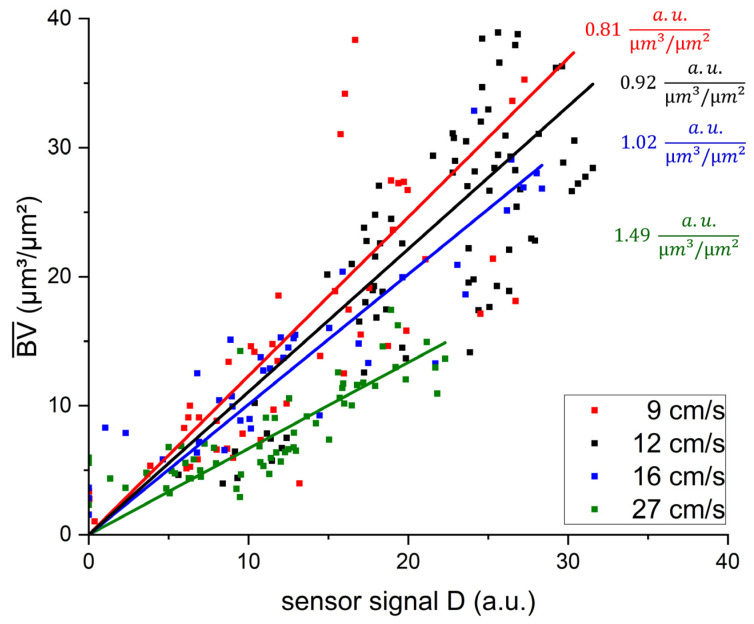
The correlation of the sensor signal with the accumulated mean biovolume ($\bar{BV}$) at the position of the sensor on the C-PP substratum in the mesofluidic flow cells. C-scans with an imaging volume of 4 × 6 × 1 mm^3^ were analyzed. The acquired biovolume was correlated with the sensor signal at the time of imaging in the respective flow cell. The results of experiments 1–4 (compare Table 1), with a mean flow velocity of 9 cm/s (red), 12 cm/s (black), 16 cm/s (blue) and 27 cm/s (green), are shown. The slopes of the correlation and the coefficients of determination R^2^ for the respective flow velocities are listed in Table 2.

For the application of the biofilm sensor as an on-line monitoring tool, its response to external factors needed to be characterized. Given the dependency of heat transfer on the hydrodynamic conditions in a system, an impact of flow velocity on sensor signal was expected. Figure 4 displays the correlation of the biofilm sensor signal with the biovolume quantified from OCT C-scans in the mesofluidic flow cells. For all of the investigated flow velocities, the correlation between accumulated biovolume and sensor signal can be described with a linear regression curve. The resulting inverse slope of the linear fit describes the sensitivity (µm^3^/(µm^2^ × a.u.)) of the biofilm sensor with the applied flow velocity (compare Equation (8)). The obtained slopes of the linear fit, sensitivities and respective coefficients of determination (R^2^) are listed in Table 2. All linear regression curves achieved coefficients of determination of 0.89 or higher, suggesting an improved correlation in comparison to the measurements performed with the C-PP or SST pipe sensors by Netsch et al. [25], with an R^2^ of 0.81 and 0.82, respectively. Due to the more frequent in situ observation of biofilm accumulation, by means of OCT, directly at the point of sensor measurement in the flow cells, heterogenous biofilm distribution on the substratum could be excluded as an influencing factor of the correlation between sensor signal and biovolume.

Furthermore, the biofilm sensors integrated into the flow cells responded with an improved sensitivity to biofilm accumulation in comparison to the pipe sensors. While the flow cell sensors identified the accumulated biofilm with a sensitivity in the range of 1 µm^3^/(µm^2^ × a.u.), the sensitivity of the SST pipe sensors was in the range of 10 µm^3^/(µm^2^ × a.u.). Generally, a lower value of sensitivity is more favorable for application of the sensor, due to more precise differentiation between different accumulated biovolumes. The determined sensor sensitivities in this study are in agreement with the sensitivity of 1.4 µm/a.u. found by Pratofiorito et al. [26] when applying the same biofilm sensor to a planar stainless steel substratum.

Additionally, the sensor sensitivity of the flow cell sensors displayed an improved response with increasing flow velocity, from 1.23 µm^3^/(µm^2^ × a.u.) at a flow velocity of 9 cm/s, to 0.67 µm^3^/(µm^2^ × a.u.) at 27 cm/s. This suggests an impact of the hydrodynamic conditions on the ratio of the thermal resistance attributed to the biofilm (*R_F_*), to the overall thermal resistance (*R_Total_*) of the heat transfer. A higher ratio results in an improved sensitivity of the sensor. While the mean biovolume accumulated on the substratum had a predominant effect on the thermal resistance, other morphological biofilm parameters may have had an impact on the thermal resistance of the biofilm and resulted in a deviation from the linear correlation between sensor signal and mean biovolume.

### 3.2. Analysis of Biofilm Morphology

**Figure 5:**
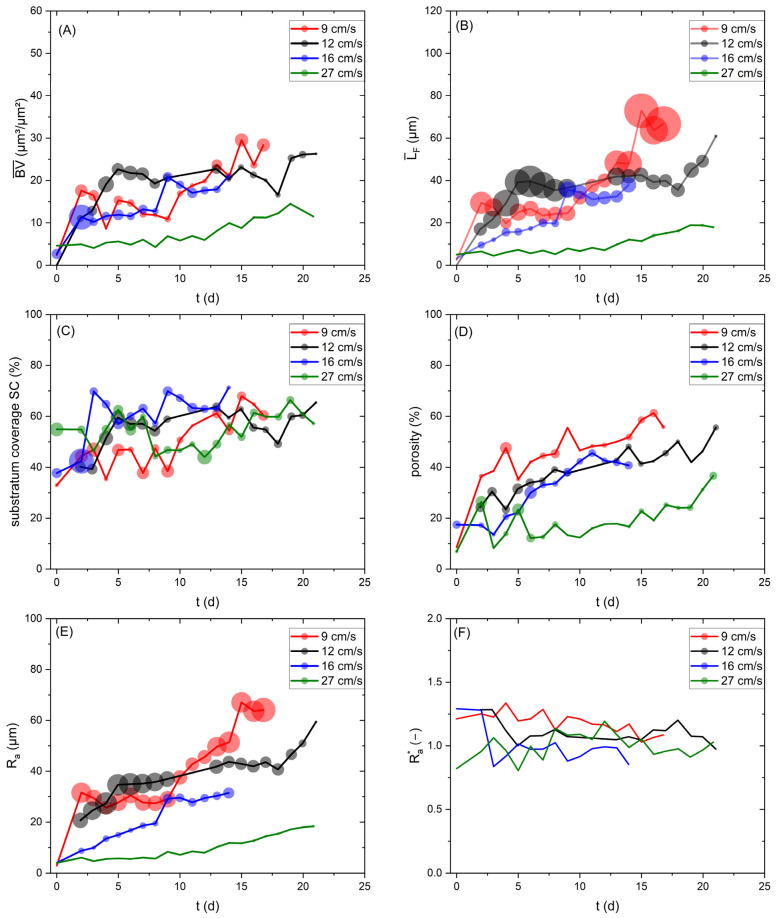
The development of the biofilm parameters over a period of up to 21 days, for cultivation at a linear flow velocity of 9–27 cm/s: (**A**) mean biovolume, (**B**) mean biofilm thickness, (**C**) substratum coverage, (**D**) porosity, (**E**) roughness and (**F**) roughness coefficient. The data points displayed are the mean value of triplicates of the respective experiments. The size of the data point shows the standard deviation.

The structural biofilm parameters are highly dependent on the hydrodynamic conditions in the cultivation system. Local distribution of shear forces and nutrient supply can result in large deviations in biofilm morphology. While the impact of hydrodynamic conditions on biofilm morphology is discussed extensively elsewhere [35,36], within this study, it is of interest as to whether variations in the morphological biofilm parameters other than the biovolume influence the heat transport from the heater to the bulk phase, and consequently impact the sensor signal. Additional morphological biofilm parameters were calculated from the 3D-OCT scans. Their development over the cultivation period for the different investigated flow velocities are shown in Figure 5.

Figure 5A shows that the mean biovolume ($\bar{BV}$), and Figure 5B shows that the mean biofilm thickness ($\bar{L}$_F_), developed similarly for the investigated flow velocities (9, 12 and 16 cm/s), resulting in a mean biofilm thickness of approx. 40 µm on Day 14. In contrast, the accumulation of biofilm at 27 cm/s shows a reduced mean biofilm thickness of up to 12 µm after 20 days of cultivation. Higher shear forces led to a decreased initial setting of bacteria, as well as increased erosion, resulting in lower biofilm accumulation. Similarly, Stoodley et al. [37] showed the development of thinner biofilms at higher flow velocities. On the contrary, Recupido et al. [38] found the development of thicker biofilms with a higher flow velocity. The impact of biovolume on the sensor signal is displayed in Figure 4. Due to the similar trends of mean biofilm thickness and biovolume, the impact of both parameters on the sensor signal is expected to be identical. The substratum coverage (*SC*) describes the percentage of the area of the sensor covered by biofilm, and thereby contributing towards a reduction in the overall thermal resistance. As shown in Figure 5C, a similar development for all experiments was found, with a substratum coverage in the range of 60–70% after 14 days of cultivation. The roughness (*R_a_*) (Figure 5E) showed a decreasing trend, with the increasing flow velocity indicating a more heterogeneous biofilm structure at lower flow velocities. Exemplary height maps from the OCT scans at different flow velocities, displaying the bulk–biofilm interface, are presented in the Supplementary Information (see Figures S1 and S2). At Day 14 of the cultivation, roughness values of 51 µm (9 cm/s), 43 µm (12 cm/s), 31 µm (16 cm/s) and 12 µm (27 cm/s) were measured.

A higher roughness of the biofilm correlates with an increased bulk–biofilm surface area, which enhances the heat transfer from the biofilm into the bulk phase. However, the roughness coefficient, describing the roughness related to the mean biofilm thickness, did not show a clear trend. For all velocities, the roughness coefficient (Figure 5F) was approx. 1. The porosity of the biofilm was the highest at 9 cm/s, while 12 cm/s and 16 cm/s showed similar developments (see Figure 5D). The lowest porosity was detected at 27 cm/s, indicating a more compressed biofilm, likely as a consequence of the higher shear forces. In porous materials, the effective thermal conductivity is a result of the difference between the thermal properties of the solid materials (EPS) and the fluid (water) in the pores. With increasing porosity, the effective thermal conductivity of the biofilm will converge towards the thermal properties of the fluid filling the pores [39,40].

The thermal conductivity of a biofilm is commonly assumed to be between 0.5 and 0.7 W/(m × K) [41], as the biofilm usually consists of approx. 90 wt.-% water, with a thermal conductivity of 0.6 W/(m × K) [31], although increases in the effective conductivity of biofilms have been measured depending on the solid content and chemical nature of the biomass. More compact biofilms with a higher fraction of inorganic compounds have increased thermal conductivities [42]. The variation in the porosity measured at the different flow velocities may have led to an increased or decreased effective thermal conductivity of the biofilm, thereby impacting the sensor signal.

Generally, the analysis of biofilm parameters is susceptible to large variations, and requires large sample sizes for statistically strong data interpretation. Gierl et al. [43] extensively discussed the variability of biofilm parameters (including mean thickness, substratum coverage and porosity) with a series of 24 replicas, showing that individual data points might significantly deviate from the real statistical mean value. As displayed in Figure 5, the size of the data points shows the standard deviation of the calculated biofilm parameters. Notably, the largest variation was visible for the biofilm parameters $\bar{L}$*_F_* and *R_a_*, while $\bar{BV}$, *SC* and porosity displayed decreased fluctuation. Moreover, the roughness coefficient was not subject to large variations. Recupido et al. [38] similarly noted that the roughness coefficient showed the smallest variation for different hydrodynamic conditions and among replicas. It seems that the variation in the biofilm parameters decreased with increasing flow velocity, as the highest flow velocity displayed the lowest fluctuation. In conclusion, we can infer that fluctuations in morphological biofilm parameters may cause a deviation from the linear correlation between mean biovolume and heat transfer resistance, thus impacting the sensor signal.

### 3.3. Reproducibility of Results

**Figure 6:**
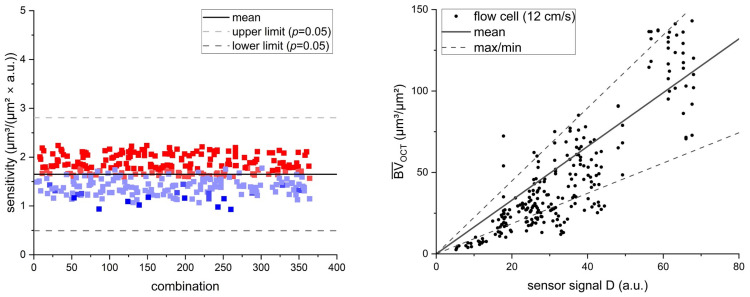
(**Left**) The calculated sensor sensitivities for the recombined flow cell combinations (triplicates) at a linear flow velocity of u = 12 cm/s. The datapoints are colored and displayed as a heatmap, according to the maximum range of the mean biovolume. Warmer-colored points (red) include a higher maximum mean biovolume than colder-colored points (blue). The gray dashed lines indicate the lower and upper limits of deviation from the mean value (*p* = 0.05). (**Right**) A display of all the data points from the 14 investigated flow cells, with the mean derived sensitivity, as well as the maximum and minimum sensitivity, calculated from the triplicate recombination.

Taking the fluctuation of the morphological biofilm parameters into account, the reproducibility of the correlation of the sensor signal with the mean biovolume requires analysis. To investigate the reproducibility, experiment 2 (u_exp_ = 12 cm/s) was repeated with a total number of investigated flow cell replicas of *n* = 14. For a comparison with experiments 1 (u_exp_ = 9 cm/s), 3 (u_exp_ = 16 cm/s) and 4 (u_exp_ = 27 cm/s) performed as triplicates, the 14 datasets acquired from experiment 2 were randomly recombined in sets of three, as they were equal probable for the sensor measurement. For the resulting 364 combined datasets, the sensor sensitivity was calculated according to the linear correlation of the sensor signal with the mean biovolume. Figure 6 Left shows the resulting sensor sensitivities derived from the linear regression of flow cell combinations.

The mean sensor sensitivity obtained from the 364 combinations revealed a less favorable sensitivity of 1.65 ± 0.31 µm^3^/(µm^2^ × a.u) compared to the initially obtained sensitivity of the initial triplicate of 1.09 µm^3^/(µm^2^ × a.u.) (see Table 2). With the larger sample size (*n* = 14), an increased fluctuation of the mean biovolume to sensor signal ratio was visible, deviating from the linear regression curve. This could indicate the influence of other morphological biofilm parameters varying over the course of the biofilm cultivation (see Figure 5). Nevertheless, the sensitivity of the recombined triplicates showed good reproducibility, as the performance of Grubb’s test for outliers revealed no significant (*p* < 0.05) outliers. This supports a sufficiently precise determination of the sensor sensitivity with triplicate experiments. Additionally, the larger sample size enabled the measurement of an increased range of biofilm thicknesses displayed in the 14 flow cells. Biofilms with a larger mean biovolume range of up to 150 µm^3^/µm^2^ were cultivated in the flow cells. In Figure 6 Left, the datapoints are colored and displayed as a heatmap, according to the range of mean biovolume included in the dataset. Larger ranges of mean biovolume are displayed with a warmer color (red), while a smaller range of biovolume is displayed with a colder color (blue). The heatmap shows that triplicate combinations with a smaller range of the mean biovolume resulted in an improved sensitivity compared to those triplicates including larger ranges of mean biovolumes. For example, the triplicate combination with the “worst” sensitivity of 2.24 µm^3^/(µm^2^ × a.u.) included a mean biovolume of up to 156 µm^3^/µm^2^, while the triplicate combination with the “best” sensitivity of 0.93 µm^3^/(µm^2^ × a.u.) only had a mean biovolume range of up to 41 µm^3^/µm^2^. This hints at a deviation from the linear correlation between mean biovolume and sensor signal for thicker biofilms, although the linear regression showed an R^2^ of 0.9 for a mean biovolume range of up to 150 µm^3^/µm^2^ (see Figure 6 Right).

### 3.4. Mathematical Model for Calculation of Sensor Sensitivity

The results obtained in this study indicate that the sensitivity of the heat transfer biofilm sensor is dependent on the hydrodynamic conditions and the geometrical properties of the substratum to which the sensor is attached. Therefore, a mathematical model to predict the sensitivity of the sensor, based on the hydrodynamic and geometrical properties, was developed. Equations and terminology for the model were taken from the VDI Heat Atlas [41]. Assuming a homogeneous distribution of the biofilm, with a porosity = 0% and full substratum coverage (*SC* = 100%) of the measurement area of the sensor, the mean biovolume ($\bar{BV}$) equals the biofilm thickness ($\bar{L}$*_F_*). As the sensor measures the change in the required heat input $\dot{Q}$ to maintain a constant temperature difference (Δ*T*) between the temperature of the medium and of the heater, the required heat input is calculated according to Equation (9). With increasing biofilm accumulation, the total thermal resistance for the heat transfer increases, thereby, the sensitivity of the sensor can be related to the change in the thermal resistance of the biofilm (*R_F_*) as a fraction of the total thermal resistance (*R_Total_*) (Equation (10)). In Equation (11), the calculation of the different fractions of the thermal resistance for the planar geometry of the mesofluidic flow cells is displayed. The total thermal resistance comprises the thermal resistance of the substratum ($R_{substatum})$, calculated from the thickness of the substratum s_C-PP_ and its thermal conductivity $\lambda_{C\text{-}PP}$; of the biofilm ($R_{F})$, from the mean biovolume and its thermal conductivity $\lambda_{F}\;$ as well as of the thermal boundary layer ($R_{tbl}$). The heat transfer coefficient α can be determined from the Nusselt number, which is dependent on the hydrodynamic conditions (compare Table 1). Given the long measurement interval of the sensor (5 min), the temporal fluctuations of the hydrodynamic conditions could be averaged for the model. More details on the calculation of the individual terms of the thermal resistance for the different geometric properties and hydrodynamic conditions can be found in Appendix A.

(9) $$\dot{Q}=\frac{∆T}{R_{Total}}$$

(10) $$sensor\;sensitivity=\frac{∆\bar{BV}}{∆\frac{R_{F}}{R_{Total}}}$$

(11) $$with\;R_{Total}=\frac{1}{A_{sensor}}\times \left(\underset{R_{substratum}}{\underbrace{\frac{s_{C\text{-}PP}}{\lambda_{C\text{-}PP}}}}+\underset{R_{F}}{\underbrace{\frac{\bar{BV}}{\lambda_{F}}}}+\underset{R_{tbl}}{\underbrace{\frac{1}{\alpha }}}\right)$$

**Figure 7:**
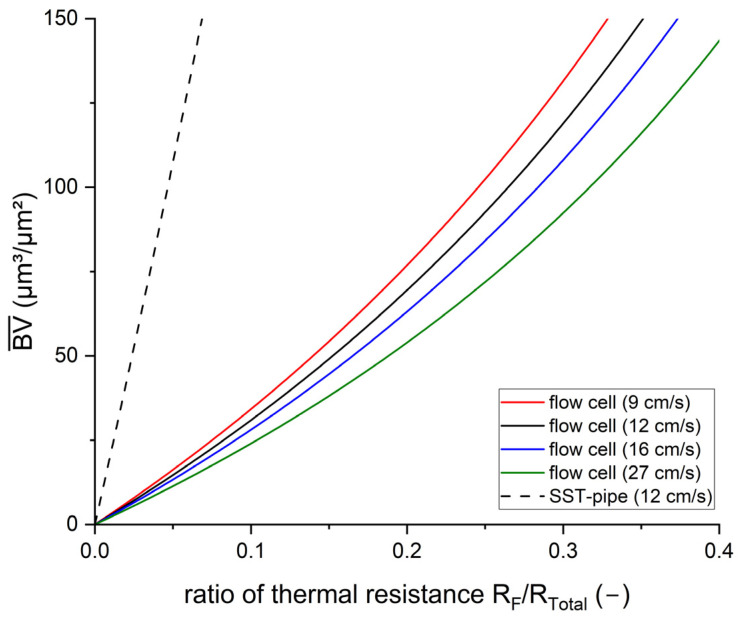
Theoretical calculation of the ratio of the biofilm’s thermal resistance compared to the total thermal resistance of the system. The calculation was performed for the SST pipe with a linear flow velocity of 12 cm/s, and for the four investigated flow velocities (9–27 cm/s) in the mesofluidic flow cells with a C-PP substratum. Details of the calculation are provided in Appendix A.

Figure 7 shows the development of the ratio of thermal resistance of the biofilm to the total thermal resistance for a biovolume of up to 150 µm^3^/µm^2^, based on the mathematical model. The model was applied for the mesofluidic flow cells with a planar C-PP substratum for the mean flow velocities of 9, 12, 16 and 27 cm/s, respectively, as well as for the SST-pipe with the mean flow velocity of 12 cm/s, investigated by Netsch et al. [25]. The steepness of the resulting curve relates to the sensitivity of the sensor, whereas a flatter gradient is more desirable for sensor application since it displays a higher resolution for the *R_F_*/*R_total_* ratio per increment of biofilm thickness.

For example, a 100 µm thick biofilm contributes to the total thermal resistance by 4.7% in the stainless steel pipe (u = 12 cm/s), while for the planar flow cells with a C-PP substratum, the fraction was 26.5% for the mean flow velocity of 12 cm/s, showing an approx. 6-fold increase. In comparison, the sensor applied to the SST-pipe achieved a sensitivity of 11 µm^3^/(µm^2^ × a.u.), as the sensor integrated into the flow cell had a mean sensitivity of 1.65 µm^3^/(µm^2^ × a.u.), resulting in an approx. 6-fold increase. Similarly to this study, Boukazia et al. [30] investigated geometrical impacts on heat flux by simulating biofouling with different layer thicknesses of PVC scotch on a flat Micro-Electro-Mechanical-System (MEMS) sensor and a cylindrical intrusive sensor. Although the sensitivity of the sensor was not reported, they found strong differences in the thermal resistance due to the geometry of the sensor application, with lower limits of detection for the flat MEMS structure. Additionally, the difference in the sensor sensitivity for the different hydrodynamic conditions in the flow cells can be seen in Figure 7. With increasing flow velocity, the convective fraction of the thermal resistance decreases, with a thinner thermal and hydrodynamic boundary layer. Consequently, with decreasing total thermal resistance, the impact of accumulating biofilm increases, and thereby the sensor sensitivity increases. Furthermore, the regression curves generally show a linear trend for the correlation between the sensor signal and biofilm accumulation (Figure 7). The theoretical calculation suggests a non-linear correlation of the sensor sensitivity with the thickness of the biofilm. The maximum biofilm thickness that was measured in the flow cells was approx. 150 µm. Within this range of biofilm thickness, the deviation of the theoretical model from the linear trend is negligible. A linear fit of the theoretical model results for the range of biofilm thickness results in an R^2^ of 0.99. The non-linearity of the sensor sensitivity is corroborated by Filladreau et al. [44], who state that the temperature difference will asymptotically reach a constant value with increasing biofilm thickness when applying a constant heat flux (compare to converted Equation (9)).

### 3.5. Comparison with Available Industrial Biofilm Sensors

Biofilm sensors must be well chosen for their target application to provide relevant information about the state of the biofilm [19]. In the case of BESs, though, commercially available electrochemical biofilm monitoring devices (e.g., ALVIM [20], BIOX [22], or BioGeorge [21]) may have a lower limit of detection; already measuring the initial bacterial layer (~1% substratum coverage [20]), they are limited in their upper limit of detection to a few µm. Therefore, they are able to provide information on the substratum coverage, but are not suitable for continuous monitoring of multilayer biofilms. In contrast, the heat transfer biofilm sensor in this study lacks the ability to identify an individual biofilm parameter such as substratum coverage. Optical measurement methods on the other hand meet the requirements for the measurement range of biofilm thickness, being able to detect medium-thick biofilms (approx. 100 µm). For example, the commercially available sensor OPTIQUAD [24] can detect thin biofilms of 1–50 µm, while optical coherence tomography (OCT) can detect biofilms in the thickness range of several 100 µm, with a resolution of a few µm [33]. Additionally, more sophisticated optical detection methods (e.g., OCT) allow for more detailed analysis of biofilm morphology, while heat transfer sensors are limited to a single biofilm parameter (e.g., mean biovolume). Nevertheless, optical methods require the installation of an optical window, which might be feasible in lab-scale reactors, though with larger increasingly complex reactors, the integration of optical windows that enable a representative measurement of the biofilm at the point of interest would be troublesome. Vibration-based biofilm sensors might present an equably viable option for biofilm monitoring compared to the heat transfer sensor presented in this study. The ultrasound-based Solenis OnGuard 3B analyzer was found to be able to measure medium-thick biofilms of up to 200 µm, with an accuracy of down to 5 µm. However, the thin biofilms in the early growth phase could only be detected with a coupled heat transfer sensor [23]. A similar detection range of 50–250 µm was reported by Maurício et al. [45] for their ultrasound-based sensor, though at an inferior limit of detection to the heat transfer sensor. The comparison with other commercially available biofilm sensors for industrial-scale application supports the use of heat transfer-based sensors for the monitoring of biofilms on the electrodes of BESs. Although, in this study, the waste water biofilm accumulated in the mesofluidic flow cells was non-electroactive, the determined sensitivities of the sensor should be transferable, as there is no expected difference in this biofilm’s thermal properties compared to those of an electroactive biofilm. With the attachment of the biofilm sensor to a planar carbonaceous substratum, the high sensitivity would allow for detailed monitoring within the optimal range of biofilm thickness (approx. up to 100 µm). It should be noted, given the dependency of the sensor sensitivity on the flow velocity found in this study, that coupling of the sensor with flow meters is required.

## 4. Conclusions

Within this study, the main objective was to characterize the impact of different hydrodynamic conditions on the sensitivity of the heat transfer biofilm sensor, as well as the application of the sensor to planar and curved substratum geometries. The integration of heat transfer biofilm sensor into mesofluidic flow cells allowed for non-invasive calibration of the dimensionless sensor signal (a.u.) to biofilm accumulation (µm^3^/µm^2^) by means of OCT. Biofilm morphology and sensor signal were measured with a high temporal resolution (1–2 days) at different biofilm ages throughout the experiments. The development of the sensor signal increased with accumulating biofilm at the point of measurement of the sensor, due to the increased thermal resistance of the biofilm. The key findings can be summarized as follows:

- While the predominant effect on the thermal resistance of the biofilm, and thus on sensor signal development, was identified as the mean biofilm thickness or biovolume, other morphological parameters (porosity, roughness, substratum coverage) might have had an impact on the thermal properties of the biofilm. This could have led to a scattering of the biofilm thickness to sensor signal ratio along a quasi-linear correlation.
- The sensitivity of the sensors in the flow cells improved due to the increased thermal resistance of the planar geometry of the C-PP substratum, despite the higher thermal conductivity of the C-PP material. The sensitivity was 6 times better than the sensor sensitivity in the SST-pipe, and 30 times better than in the C-PP pipe.
- With increased flow velocities (more turbulent hydrodynamic conditions), the sensitivity of the sensor increased from 1.26 µm^3^/(µm^2^ × a.u.) at 9 cm/s, to 0.67 µm^3^/(µm^2^ × a.u.) at 27 cm/s, due to the decreased thickness of the thermal boundary layer. For precise conversion, the heat transfer biofilm sensor signal must be coupled with a measurement of the flow velocity.
- A mathematical model of the biofilm sensor, incorporating hydrodynamic effects and geometrical heat transfer regimes, was developed. The model can support the prediction of the sensitivity of biofilm sensors for various applications.

The applicability of the heat transfer biofilm sensor to a planar C-PP substratum with an improved sensitivity, in the range of a few µm^3^/(µm^2^ × a.u.), compared to stainless steel pipes, enables the installation of the sensors in situ into the electrodes of BESs, and thereby directly at the point of interest in the biofilm reactor, under the same hydrodynamic conditions as electroactive biofilm. The high sensitivity of the biofilm sensor is comparable to that of laboratory optical measurement techniques such as OCT, and enables the precise monitoring of the electroactive biofilm in the assumed optimal range of biofilm thickness of up to 100 µm.

## Figures and Tables

**Table 1:** An overview of the experimental conditions. Each replica consisted of one SST pipe sensor, one C-PP pipe sensor and a mesofluidic flow cell with a biofilm sensor installed on the C-PP substratum.

Experiment	Q (L/min)	u_flow cell_ (cm/s)	u_pipe_ (cm/s)	No. of Replicates
1	1.94	9 (Re = 1320)	6.39 (Re = 1620)	3
2	2.6	12 (Re = 1980)	8.56 (Re = 2430)	14
3	3.46	16 (Re = 2350)	11.38 (Re = 2880)	3
4	5.83	27 (Re = 3970)	19.18 (Re = 4850)	3

**Table 2:** The sensitivity of the biofilm sensor, calculated from the linear regression of the correlation between mean biovolume and sensor signal from experiments 1–4 (compare Table 1).

Flow Velocity (cm/s)	Slope of Linear Correlation (a.u. × (µm^3^/µm^2^))	Sensitivity (µm^3^/(µm^2^ × a.u.))	Coefficient of Determination R^2^	Range of Measured Biovolume (µm^3^/µm^2^)
9	0.81	1.23	0.89	0–38
12	0.92	1.09	0.94	0–39
16	1.02	0.98	0.95	0–33
27	1.49	0.67	0.93	0–18

## Data Availability

The original contributions presented in this study are included in the article/supplementary material. Further inquiries can be directed to the corresponding author.

## Supplementary Materials

The following supporting information can be downloaded at https://www.mdpi.com/article/10.3390/bios15020093/s1: Figure S1: Height maps (6 × 4 mm^2^) displaying the bulk-biofilm interface of the flow cells A, B and C for all four flow velocities at Day 7 of the cultivation. The direction of flow was from left to right. Figure S2: Height maps (6 × 4 mm^2^) displaying the bulk-biofilm interface of the flow cells A, B and C for all four flow velocities at Day 14 of the cultivation. The direction of flow was from left to right.

## Abbreviations

The following abbreviations are used in this manuscript:

BES	Bioelectrochemical systems
C-PP	Carbon–polypropylene
EET	Extracellular electron transfer
MEC	Microbial electrolysis cell
MEMS	Micro-Electro-Mechanical-System
MFC	Microbial fuel cell
OCT	Optical coherence tomography
POM	Polyoxymethylene
PUR	Polyurethane
PVC	Polyvinylchloride
SC	Substratum coverage
SST	Stainless steel
TBL	Thermal boundary layer

## Appendix A. Model for Theoretical Calculation of Sensor Sensitivity

For the development of the theoretical model of sensor sensitivity, equations from the VDI Heat Atlas were used to calculate the three different contributors to the thermal resistance.

**Table A1:** The input parameters for the mathematical model.

**Geometric Variables**		
$A_{Sensor}$	Sensor area	100 mm^2^
$d_{i}$	Inner diameter of pipe	25.4 mm
$l_{pipe}$	Length of pipe	250 mm
$l_{Sensor}$	Length of sensor area	10 mm
$r_{i}$	Inner radius of pipe	12.7 mm
s	Wall thickness of pipe	3 mm
$s_{C\text{-}PP}$	Wall thickness of C-PP	4 mm
$\bar{BV}$ $\;\mathrm{or}\;{\bar{L}}_{F}$	Mean biovolume or mean biofilm thickness	0–1000 (µm^3^/µm^2^) or (µm)
**Thermodynamic variables**		
α	Heat transfer coefficient	W/(m^2^ × K)
$\dot{Q}$	Heat flux	W
R	Thermal resistance	K/W
∆T	Temperature difference	10 K
**Material properties at 25 °C**		
$\lambda_{C\text{-}PP}$	Thermal conductivity of C-PP	21 W/(m × K)
$\lambda_{F}$	Thermal conductivity of biofilm	0.6 W/(m × K)
$\lambda_{water}$	Thermal conductivity of water	0.6 W/(m × K)
$\lambda_{SST}$	Thermal conductivity of SST	13.6 W/(m × K)
**Dimensionless numbers**		
Nu	Nusselt number	f(Re, Pr)
Re	Reynolds number	Compare Table 1
Pr	Prandtl number	6.137
ζ	Turbulence factor	-
γ	Intermittence factor	-

The required heat input $\dot{Q}$ to maintain a constant temperature difference ∆*T* is calculated as follows:

(A1) $$\dot{Q}=\frac{∆T}{R_{Total}}$$

with the following:

(A2) $$R_{Total}=R_{substatum}+R_{F}+R_{tbl}$$

The sensor sensitivity relates to the following:

(A3) $$sensor\;sensitivity≅\frac{∆\bar{BV}}{∆\frac{R_{F}}{R_{Total}}}$$

For the application of the sensor to a planar substratum (e.g., flow cell), the following calculations are used:

(A4) $$R_{substratum}=\frac{1}{A_{Sensor}}\frac{s_{C\text{-}PP}}{\lambda_{C\text{-}PP}}$$

(A5) $$R_{F}=\frac{1}{A_{Sensor}}\frac{{\bar{L}}_{F}}{\lambda_{F}}$$

(A6) $$R_{tbl}=\frac{1}{A_{Sensor}\times \alpha }$$

The heat transfer coefficient α is a function of the Nusselt number (Nu = f (Re,Pr)), as follows:

(A7) $$\alpha_{fc}=\frac{{Nu}_{fc}\times \lambda_{water}}{l_{Sensor}}$$

For flow conditions 10 < *Re* < $10^{7}$, the following calculation is used:

(A8) $${Nu}_{fc}=\sqrt{{Nu}_{lam}^{2}+{Nu}_{turb}^{2}}$$

with

(A9) $${Nu}_{lam}=0.664\times {Re}^{\frac{1}{2}}\times {Pr}^{\frac{1}{3}}$$

(A10) $${Nu}_{turb}=\frac{0.037\times {Re}^{0.8}\times Pr}{1+2.443\times {Re}^{-0.1}\times ({Pr}^{\frac{2}{3}}-1)}$$

For the application of the sensor to a cylindrical substratum (e.g., pipe), the following calculations are used:

(A11) $$R_{substratum}=\frac{1}{{2\times \pi \times l}_{Sensor}}\times \frac{\ln(\frac{r_{i}+s}{r_{i}})}{\lambda_{SST}}$$

(A12) $$R_{F}=\frac{1}{{2\times \pi \times l}_{Sensor}}\times \frac{ln\left(\frac{r_{i}}{r_{i}-{\bar{L}}_{F}}\right)}{\lambda_{F}}$$

(A13) $$R_{tbl}=\frac{1}{A_{Sensor}\times \alpha }$$

The heat transfer coefficient α is a function of the Nusselt number (Nu = f (Re,Pr)), as follows:

(A14) $$\alpha_{pipe}=\frac{{Nu}_{pipe}\times \lambda_{water}}{d_{i}}$$

For laminar flow conditions *Re* < 2300, the following calculation is used:

(A15) $${Nu}_{pipe}=0.332\times {Pr}^{\frac{1}{3}}\times ({Re\times \frac{d_{i}}{l_{pipe}})}^{\frac{1}{2}}$$

For flow conditions in the transient zone 2300 < *Re* < $10^{4}$, the following calculation is used:

(A16) $${Nu}_{pipe}=\left(1-\gamma \right)\times {Nu}_{pipe,lam}+{\gamma \times Nu}_{pipe,\;turb}$$

with the intermittence factor γ calculated as follows:

(A17) $$\gamma =\frac{Re-2300}{10^{4}-2300}$$

(A18) $${Nu}_{pipe,turb}=\frac{\frac{\zeta }{8}\times 10^{4}\times Pr}{(1+12.7\sqrt{\frac{\zeta }{8}\times ({Pr}^{\frac{2}{3}}-1)}}\times \left(1+\frac{1}{3}\times {\left(\frac{d_{i}}{l_{sensor}}\right)}^{\frac{2}{3}}\right)$$

with the turbulence factor ζ calculated as follows:

(A19) $$\zeta ={\left(1.8\times \log\left(Re\right)-1.5\right)}^{-2}$$

(A20) $${Nu}_{pipe,lam}={\left(49.371+{{(Nu}_{m,t,2,lam}-0.7)}^{3}+{Nu}_{m,t,3,lam}^{3}\right)}^{\frac{1}{3}}$$

(A21) $${Nu}_{m,t,2,lam}=1.615\times {\left(2300\times Pr\times \frac{d_{i}}{l_{sensor}}\right)}^{\frac{1}{3}}$$

(A22) $${Nu}_{m,t,3,lam}={\left(\frac{2}{1+22\times Pr}\right)}^{0.18}\times {\left(2300\times Pr\times \frac{d_{i}}{l_{sensor}}\right)}^{\frac{1}{2}}$$
