# Supplementary material for: In Situ Biofilm Monitoring Using a Heat Transfer Sensor: The Impact of Flow Velocity in a Pipe and Planar System
Source: Biosensors (Basel). 2025 Feb 6;15(2):93. doi: 10.3390/bios15020093 (PMC11853227; doi:10.3390/bios15020093)
Supplement: Supplementary file 1 [file biosensors-15-00093-s001.zip › biosensors-3442862-supplementary.pdf]

Supporting Information for:

In Situ Biofilm Monitoring Using a Heat Transfer Sensor: The Impact of Flow Velocity in a Pipe and Planar System

Andreas Netsch *, Shaswata Sen, Harald Horn and Michael Wagner

This SI contains 1 page of additional information, including 2 Figures

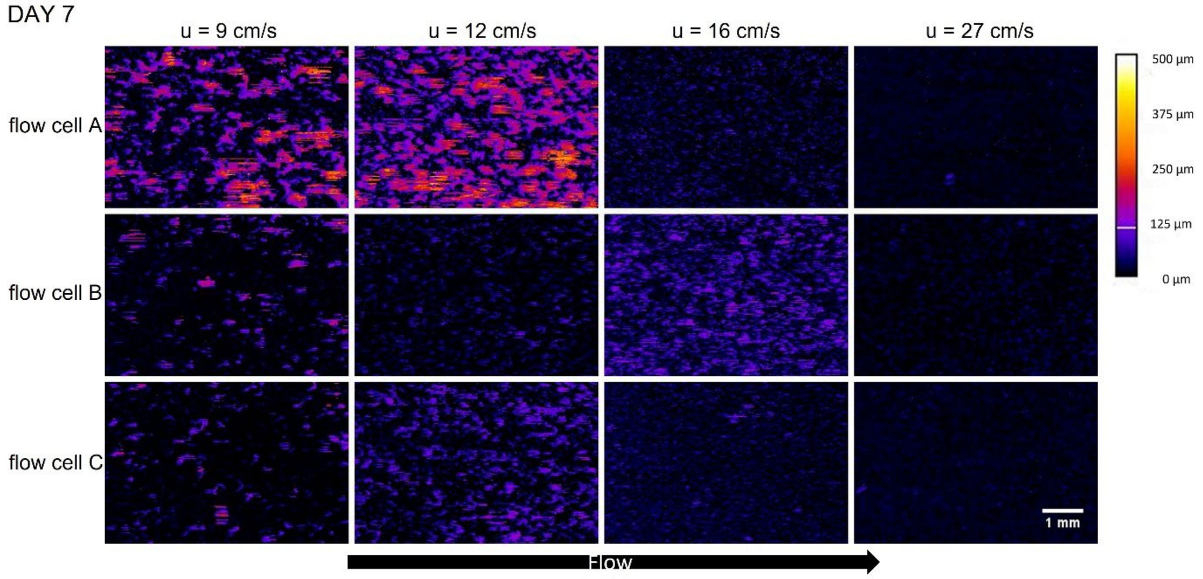

Figure S1. Height maps (6 x 4 mm^2) displaying the bulk-biofilm interface of the flow cells A, B and C for all four flow velocities at Day 7 of the cultivation. The direction of flow was from left to right.

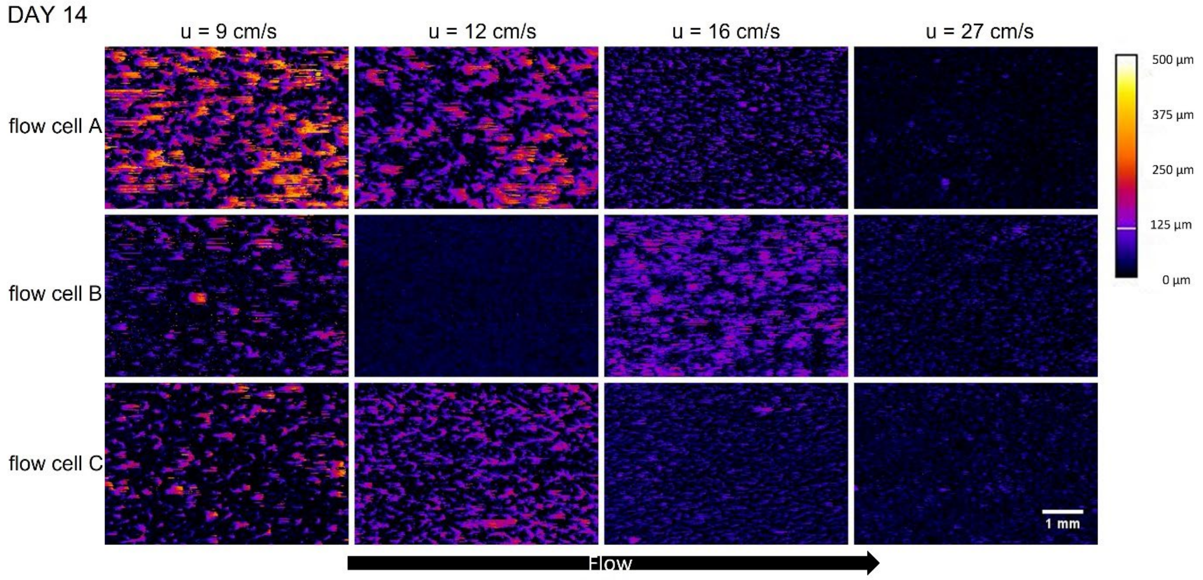

Figure S2. Height maps (6 x 4 mm²) displaying the bulk-biofilm interface of the flow cells A, B and C for all four flow velocities at Day 14 of the cultivation. The direction of flow was from left to right.
